# Supplementary material for: Chromothripsis during telomere crisis is independent of NHEJ, and consistent with a replicative origin
Source: Genome Res. 2019 May;29(5):737–49. doi: 10.1101/gr.240705.118 (PMC6499312; doi:10.1101/gr.240705.118)
Supplement: Supplemental Material [file supp_gr.240705.118_Supplemental_file_1.zip › contigs/annotated_contigs/DB113/contig.2.DB113_length_716_mean_cov_10.2234636872.docx]

**DB113_length_716_mean_cov_10.2234636872**

```
ACCTCAAGTGATCTGTCTGCTTCAGCTTCCCAAAGTGTGAGATTACAGGCATGGGCCACCATGCTCGGCCTATGTAGACAATGTCATTT
 >chr10:12358652-12359026 + E=3e-213
TAAAAAATGGAAGTAATTAAAAAGGGAAAGACCCATAGATTACATTATATAAAATGTAAAACATTTTTTGGTCAAAATATCATAAGCAA

AATTAAAACACATATTACAACAAACACCAATGAAATATATTAAGAGCTTTAAAAATATAAAACAGCTGGGCGCGGTGGCTCATGCCTGT

AATCCCAGCACTTTGGGAGGCTGAGGCGGGCGGATCACGAGGTCAGGAGATCGAGACCATCCTGGCTAACATGGTGAAACCCCGTCTCT

ACTAAAAGTACA|AAAAAT|GGCTACACAGAACTTGAGAAGGTGTGGAAGGGGACAGAATACCCTTATTCCCATTGGCTTGGGGGCTGT
             >chr10:13270569-13270916 - E=9e-193
GGGGATGGGGCAACTGTTTGCTGACTGTGTGGATATTAAACTCCTTTCCCTCCCTCCTCCTATTTGGGCATCACCGCACTGGTCTGTGT

TCTCCGTCTAAGCACCAACACTTAGGCTTAGCTCTGGCAGGATCATATTGGACAGAGTCCACACTCACTCGCAGGTGCCTTATATTCAC

GTCAATGTCCTGGCTCCAGGTCCTACTCCATCCTGGAAAATCAAGGCCTTCATTGACCTATTCAGTGTACTCAGCATCTTTACTGACTG

GCAGAA
```
